# Preoperative red cell distribution width predicts postoperative cognitive dysfunction after coronary artery bypass grafting

**DOI:** 10.1042/BSR20194448

**Published:** 2020-04-21

**Authors:** Jing Wan, Peiwen Luo, Xiaonan Du, Hong Yan

**Affiliations:** Department of Anesthesia, The Central Hospital of Wuhan, Tongji Medical College, Huazhong University of Science and Technology, Wuhan City, Hubei Province 430014, China

**Keywords:** biomarker, coronary artery bypass grafting, postoperative cognitive dysfunction, red cell distribution width

## Abstract

We assessed the relationship between red blodd cell distribution width (RDW) and postoperative cognitive dysfunction (POCD) after coronary artery bypass grafting (CABG) in patients who usually had obvious hemodynamic changes. We enrolled 362 coronary heart disease patients who received CABG. POCD was assessed through neuropsychological examination at 21 days after operation. Demographics, history of diseases, blood biochemical parameters and perioperative data were collected. The receiver operating characteristic (ROC) curve was used to find the best cut-off value of RDW for diagnosis of POCD. Logistic regression was used to explore the relationship between RDW and POCD. The 21-day incidence of POCD in patients receiving CABG was 27.1% (98/362). The RDW of POCD patients was significantly higher than in the non-POCD patients (17.4 vs. 13.2). The sensitivity and specificity of RDW for predicting POCD were 82.7 and 64.8%, respectively. The POCD patients also tended to be older and had higher fasting plasma glucose, hypersensitive c-reactive protein, tumor necrosis factor-α, white blood cell levels and longer surgery time. No significant differences were found in other parameters. The 21-day neuropsychological test results were better in the POCD patients than the non-POCD patients. After adjustment of potential factors, the preoperative high RDW was still associated with an increased risk of POCD (odds ratio (OR) = 2.52, 95% confidence interval (CI): 1.28–4.31). Our study indicates that preoperative RDW is significantly elevated in POCD patients receiving CABG. The elevated preoperative RDW is associated with an increased risk of POCD and preoperative RDW can be an independent predictor of POCD.

## Introduction

Postoperative cognitive dysfunction (POCD) is one of the most common complications after coronary artery bypass grafting (CABG) [1]. POCD comprises a wide range of cognitive functions, including long-term memory, abstract generalization ability, mental concentration and analysis of knowledge applying ability, and thus lowers quality of life and increases mortality [2]. Clinical diagnosis of POCD involves a series of neuropsychological tests. However, there is no unified standard concerning the selection of tests, main reference points and follow-up duration, leading to different prevalent rates of POCD in different institutions [3]. Therefore, systemic, effective and simple diagnostic criteria should be developed for POCD. POCD pathogenesis has not been properly clarified, but it is affirmatory that POCD is a complication of cardinal surgery induced by multiple factors. Some recognized important risk factors of POCD include the advanced age, combined metabolic diseases, operative procedure, anesthesia induction, pre-existing cerebrovascular and systemic vascular diseases, and systemic inflammation [4]. However, the risk factors vary among different studies because of the variant diagnosis of POCD, diverse analysis procedures, and subjective effects [5]. Therefore, systemic in-depth research should be performed to quest the high correlation with POCD risk factors, to underlie clinical prevention of POCD, and to establish an effective behavior evaluation method.

The red blood cell distribution width (RDW) is a simple and inexpensive indicator of erythrocyte volume heterogeneity and is traditionally used in laboratory hematology for differential diagnosis of anemias [6]. Nonetheless, recent evidence attests that anisocytosis is commonplace in human disorders, such as cardiovascular diseases [7], venous thromboembolism [8], cancer [9], and diabetes as well as other acute or chronic conditions [10,11]. An increased RDW has a high negative predictive value for diagnosing various disorders, and also conveys important information for short- and long-term prognoses. RDW is associated with a variety of cerebrovascular diseases and prognosis, such as stoke [12], cerebral infarction [13] and cerebral thrombosis [14], indicating that RDW may be involved in the cerebral hemodynamic changes. The cerebral blood flow in normal subjects is constant under different contexts and is decided by the cardiac output and the regulatory mechanism. Accumulating studies show that patients with cardiac dysfunction have lower cerebral blood flow. The decrease in cerebral blood flow will cause the disorder of cerebral auto-regulation mechanism, which may lead to cerebral degenerative abnormalities, such as cognitive dysfunction. Moreover, cognitive function changes are involved in many inflammatory response processes and oxidative stress [15,16]. RDW is considered to be a biochemical marker of pre-inflammatory state. Oxidative stress and inflammatory response can increase RDW by damaging iron metabolism and reducing erythrocyte survival through the regulation of erythrocruorin [17]. Considering the above findings, we assessed the relationship between RDW and cognitive dysfunction in patients receiving CABG who usually had obvious hemodynamic changes.

## Materials and methods

### Study population

We enrolled coronary heart disease patients who received CABG in the Central Hospital of Wuhan from August 2015 to September 2018. The inclusion criteria were: (1) diagnosis with coronary heart disease; (2) multivessel coronary artery diffuse stenosis (>75%) and needing CABG; (3) preoperative mini-mental state examination (MMSE), Self-rating Depression Scale (SDS) and Self-Rating Anxiety Scale (SAS) indicating no cognitive impairment (illiteracy > 17, primary school > 20, junior high school and above > 25) [18], depression (SDS < 53) [19] or anxiety symptoms (SAS < 50) [20]; (4) significant improvement of vessel stenosis degrees after CABG, and relief of angina pectoris and heart dysfunction. The exclusion criteria were: (1) cognitive impairment according to the preoperative MMSE; (2) severe central nervous system disease or mental illness (stroke, transient ischemic attack, ischemic attack, severe anxiety, drug addiction, alcoholics) or history of brain surgery; (3) intake of nerve or psychiatric drugs; (4) inability to receive the cognitive function assessment because of hearing/visual impairment; (5) combination with other cardiovascular diseases that required extra surgery. The present study was approved by the Ethics Committee of Tongji Medical College, Huazhong University of Science and Technology, and the patients signed the informed consent.

### Data collection

The data collection included three parts: demographics obtained via questionnaire, including age, gender, height and weight for calculating body mass index (BMI), education year, smoking and drinking status, physical activity frequency; history of hypertension, diabetes, hyperlipidemia, chronic renal dysfunction, aortic plaque, carotid artery stenosis, and cerebrovascular disease collected form medical records. Preoperatively, 5 ml of venous blood was taken from each patient within 24 h after admission for measurement of blood biochemical parameters. For the blood tests, hemogram (red blood cells, white blood cells, red cells, bold platelets, and hemoglobin) and RDW were determined from whole blood using a Sysmex XE_5000 analyzer (Sysmex Canada, Inc., Canada). The biochemical parameters were completed using a fully automatic biochemical analyzer (HITACHI, Japan), including triglyceride, high density lipoprotein-cholesterol (HDL-C), low density lipoprotein-cholesterol (LDL-C), total cholesterol, and fasting plasma glucose. Hypersensitive C reactive protein (hs-CRP) was measured using an immune turbidity method. Other perioperative parameters including duration of surgery, duration of surgery, surgery methods (off-pump, no-pump), and the baseline visual analog scale were also measured and used to assess postoperative pains and the presence of mood disorders.

### Diagnosis of PCOD

All participants experienced neuropsychological examination preoperatively and at 7 and 21 days after operation. This examination consisted of visual verbal learning test and visuospatial memory test (both including delayed recall test, discrimination index), trail-making test, Benton judgment of line orientation, digit span test, symbol-digit modalities test, and verbal fluency test. According to the international study of POCD, a Z-value was obtained for individual examination. Z-value was calculated as: Z = (postoperative score − preoperative score)/corresponding standard deviation.

A patient was diagnosed as POCD if Z-values of two or more tests were equal or greater than 1.96 [21].

### Anesthesia method and surgery

In the evening prior to surgery, patients took 0.1 mg/kg diazepam with β-blockers or nitrates maintained until surgery. All patients were routinely and intramuscularly injected with 0.1 mg/kg morphine and 0.3 mg of scopolamine at 30 min before surgery. All patients underwent routine surveillance after entering the operating room. Anesthesia was induced via injecting midazolam, etomicolus, sufentanil, and atracurium cisphenylate. Mechanical ventilation was performed after endotracheal intubation. A three-chamber central venous catheter or swan-ganz floating catheter was inserted into the internal jugular vein to monitor central venous pressure, mean pulmonary artery pressure, and pulmonary capillary embolus. Plasma target-controlled infusion of propofol was combined with inhalation of sevoflurane. Remifentanil was injected to maintain anesthesia, and atracurium cisphenylate was administered intermittently. After the operation, all patients were transferred to the intensive care unit of cardiac surgery with the tracheal tubes preserved. Two surgery methods were adopted (on-pump CABG and off-pump CABG) for different patients. This surgery was performed by skilled surgeons. All aspects of patient conditions were recorded in the medical reports.

### Statistical analysis

Statistical analyses were completed on SPSS 23.0. According to the criteria of POCD, a POCD group and a non-POCD group were set up. Continuous data were expressed as mean ± standard deviation, and compared between groups via *t* test. Category data were expressed as count and percentage, and compared between groups via Chi- square test. For other situations, the non-parameter test was used. To explore the relationship between RDW and POCD, we first plotted the receiver operating characteristic (ROC) curve to find the best cut-off value of RDW for diagnosing POCD, and calculated sensitivity and specificity. The relationship between hs-CRP and RDW was assessed via Pearson correlation analysis. Univariate and multivariate logistic regressions were performed and the corresponding odds ratios (ORs) and 95% confidence intervals (CIs) were calculated. The multivariate logistic regression only involved the variables that were significant in univariate analysis. Specifically, these variables were age, aortic plaque, carotid artery stenosis, cerebrovascular disease, HDL-C, fasting plasma glucose, hs-CRP, TNF-α, white blood cell, RDW,and duration of surgery. *P*<0.05 was considered as the significant level.

## Results

### Baseline characteristics of study participants

Finally, 362 patients met the inclusion criteria, and their general characteristics are presented in Table 1. There were 205 males (56.6%) and 157 females (43.4%). The mean age was 67.1 years. The 362 patients consisted of 153 (42.3%) smokers and 121 (57.7%) drinkers. The mean education duration was 7.1 years. The 21-day incidence of POCD among patients receiving CABG was 27.1% (98/362). The POCD patients tended to be older compared with the non-POCD group (68.2 vs. 66.7, *P*=0.037). No significant differences between groups were observed in gender ratio (*P*=0.550), BMI (*P*=0.634), education level (*P*=0.057), smoking (*P*=0.705), drinking (*P*=0.755), or physical activity (*P*=0.414). About the history of diseases, there were no significant between-group differences in prevalence rates of hypertension (*P*=0.134), hyperlipidemia (*P*=0.789), or chronic renal dysfunction (*P*=0.853). However, the prevalence of diabetes was significantly higher in the POCD group than in the non-POCD group (43.9 vs. 32.2%, *P*=0.039). Significant differences between the POCD and non-POCD groups were observed in the prevalence rates of aortic plaque (51.0 vs. 33.7%, *P*=0.003), carotid artery stenosis (40.8 vs. 28.0%, *P*=0.020) and cerebrovascular diseases (34.7 vs. 14.0%, *P*<0.001).

**Table 1:** Comparison of clinical characteristics between POCD group and non-POCD group at 21 days after operation

Parameters	POCD group (*n*=98)	Non-POCD group (*n*=264)	t/χ^2^	*P*
Demographics				
Age, year	68.2 ± 5.6	66.7 ± 6.2	2.098	0.037
Male, *n* (%)	58 (59.1%)	147 (55.7%)	0.357	0.550
BMI, kg/m^2^	23.1 ± 9.3	22.6 ± 8.7	0.477	0.634
Education, y	6.5 ± 2.8	7.3 ± 2.6	1.910	0.057
Smoking, *n* (%)	43 (43.9%)	110 (41.7%)	0.143	0.705
Drinking, *n* (%)	34 (34.7%)	87 (33.0%)	0.097	0.755
Physical activity			1.762	0.414
<1 time/week	48 (49.0%)	117 (44.3%)		
1–3 times/week	22 (22.4%)	52 (19.7%)		
>3 times/week	28 (28.6%)	95 (36.0%)		
Hypertension, *n* (%)	90 (91.8%)	227 (86.0%)	2.248	0.134
Diabetes, *n* (%)	43 (43.9%)	85 (32.2%)	4.266	0.039
Hyperlipidemia, *n* (%)	55 (56.1%)	144 (54.5%)	0.072	0.789
Chronic renal dysfunction, *n* (%)	8 (8.2%)	90 (7.6%)	0.035	0.853
Aortic plaque, *n* (%)	50 (51.0%)	89 (33.7%)	9.052	0.003
Carotid artery stenosis, *n* (%)	40 (40.8%)	74 (28.0%)	5.416	0.020
Cerebrovascular disease, *n* (%)	34 (34.7%)	37 (14.0%)	19.384	<0.001
Blood biochemical parameters				
Triglyceride, mmol/dl	1.7 ± 1.1	1.5 ± 0.9	1.765	0.078
HDL-C, mmol/dl	1.1 ± 0.3	1.2 ± 0.3	2.818	0.005
LDL-C, mmol/dl	3.2 ± 1.0	3.3 ± 0.9	0.911	0.363
Total cholesterol, mmol/dl	5.4 ± 0.9	5.2± 0.9	1.879	0.061
Fasting plasma glucose, mmol/l	6.7 ± 2.2	5.6 ± 1.2	6.058	<0.001
Hs-CRP, ng/l	9.0 ± 14.3	5.2± 11.2	2.634	0.009
Red blood cell, ×10^12^/l	4.5 ± 0.6	4.4 ± 0.5	3.197	0.111
White blood cell, ×10^9^/l	6.5 ± 2.4	5.8 ± 2.5	2.393	0.017
Red cell distribution width (%)	17.4 ± 0.2	13.2 ± 0.4	11.814	<0.001
Blood platelet, ×10^9^/l	210.6 ± 64	205.4 ± 54	0.773	0.440
Hemoglobin, g/l	132.5 ± 13.4	135.6 ± 14.8	1.815	0.070
Perioperative parameters				
Duration of surgery, h	4.3 ± 1.2	3.8 ± 0.9	4.270	<0.001
Surgery methods			0.187	0.665
Off-pump	38 (38.8%)	109 (41.3%)		
On-pump	60 (61.2%)	155 (58.7%)		
Baseline VAS	4.4 ± 0.9	4.3 ± 1.0	0.868	0.386
Baseline MMSE	25.7 ± 2.6	26.2 ± 2.3	1.773	0.077
Baseline SAS	24.6 ± 2.5	24.1 ± 2.3	1.794	0.074
Baseline SDS	24.4 ± 3.3	25.0 ± 2.8	1.723	0.086

**Figure 1:**
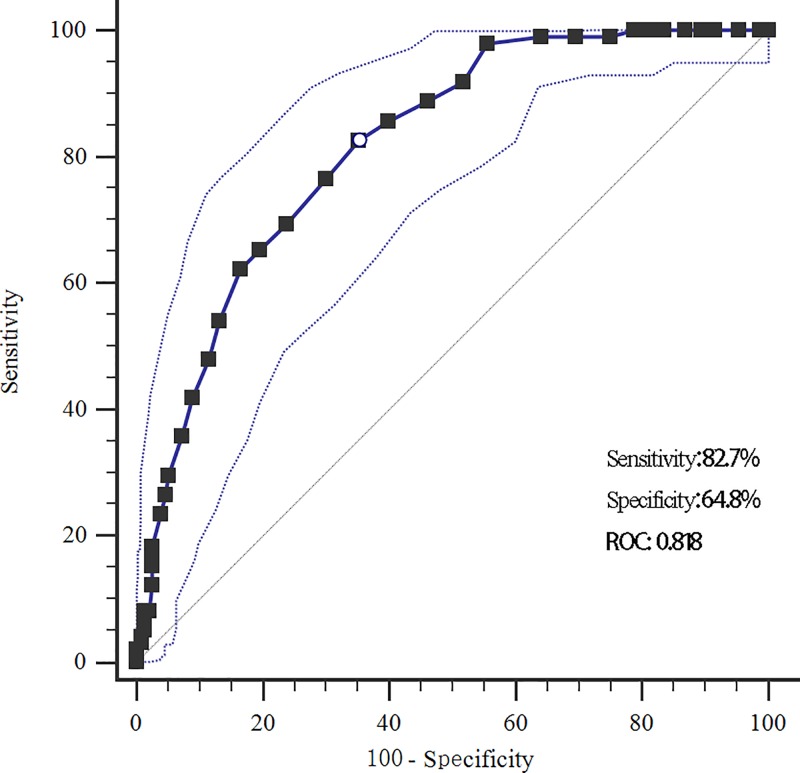
The ROC curve of red cell distribution width for diagnosing POCD (sensitivity: 82.7%, specificity: 64.8%, AUC: 0.818, cutoff: 14.7%)

**Figure 2:**
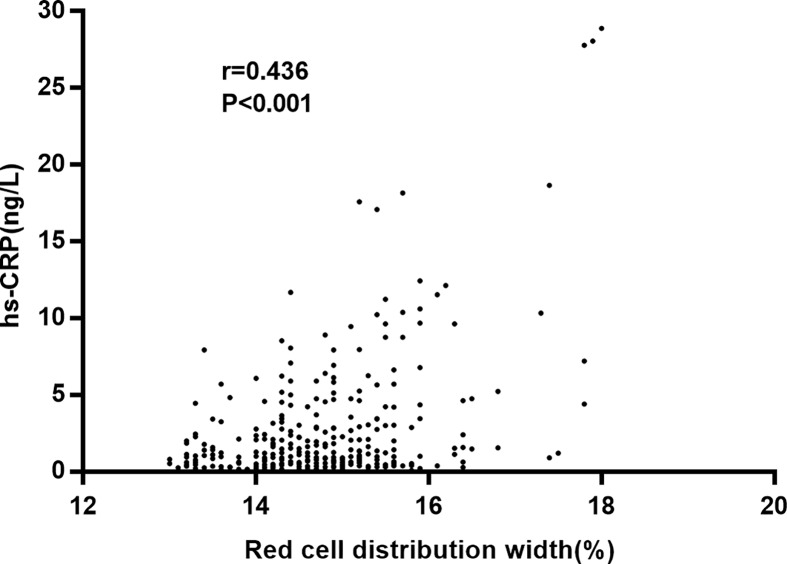
The scatter plot of the positive relationship between RDW and hs-CRP (r=0.436, *P*<0.001)

We further compared the blood biochemical parameters between groups. The RDW of the POCD group was significantly higher than that in the non-POCD (17.4 vs. 13.2, *P*<0.001). The sensitivity and specificity of RDW for predicting POCD were 82.7 and 64.8%, respectively, and the cut-off value was 14.7 (Figure 1). A positive relationship was found between RDW and hs-CRP level (r = 0.436, *P*<0.001, Figure 2). The POCD group also tended to have significantly higher levels of fasting plasma glucose (*P*<0.001), hs-CRP (*P*=0.009), TNF-α (*P*=0.025), and white blood cell (*P*=0.017), but significantly lower HDL-C level compared with the non-POCD group (*P* = 1.1 vs. 1.2, *P*=0.005). For perioperative parameters, the POCD group took longer surgery time (4.3 vs. 3.8 h, *P*<0.001) than the non-POCD group. No significant difference between groups was found in surgery methods (*P*=0.665).

### Neuropsychological test

The baseline MMSE, SAS, SDS, and VAS assessments were presented in Table 1. No significant differences were observed in these baseline parameters. Comparisons between baseline and 21-day neuropsychological test results are presented in Table 2. At the baseline level, there were no significant differences in any neuropsychological test. At 21 days after operation, the verbal leading test, visuospatial memory test, trail-making test, Benton judgment of line orientation, digit span test, symbol-digit modalities test, and discrimination index were all significantly better in the POCD group than in the non-POCD group (*P*<0.05). However, the delayed recall test, discrimination index of verbal learning-test, delayed recall test of visuospatial memory test and verbal fluency test indicated no significant differences (*P*>0.05).

**Table 2:** Comparison of neuropsychological test results at baseline and 21 days after operation

Index	Baseline			21 days after operation		
	POCD	Non-POCD	*P*	POCD	Non-POCD	*P*
Verbal learning test-revised	13.5 ± 3.1	14.1 ± 2.6	0.065	9.2 ± 3.3	10.6 ± 3.5	0.001
Visuospatial memory test-revised	6.3 ± 2.0	6.4 ± 1.8	0.649	4.2 ± 2.2	5.3 ± 2.6	0.000
Trail-making test	309.1 ± 75.6	292.5 ± 81.2	0.079	385.4 ± 99.5	333.4 ± 92.6	0.000
Benton judgment of line orientation	15.8 ± 2.5	15.5 ± 2.7	0.339	10.8 ± 2.8	12.5 ± 2.5	0.000
Digit span test	16.3 ± 2.6	15.9 ± 2.9	0.231	18.8 ± 3.2	14.9 ± 3.0	0.003
Symbol-digit modalities test	17.5 ± 3.8	17.6 ± 4.5	0.845	13.7 ± 4.8	15.4 ± 4.3	0.001
VLT-R delayed recall test	3.6 ± 1.2	3.8 ± 1.5	0.236	2.8 ± 1.8	3.1 ± 1.5	0.111
VLT-R discrimination index	23.1 ± 1.2	22.8 ± 1.5	0.076	21.3 ± 2.2	21.7 ± 1.7	0.068
VMT-R delayed recall test	2.9 ± 1.0	3.0 ± 1.1	0.432	2.2 ± 1.4	2.3 ± 1.9	0.635
VMT-R discrimination index	11.0 ± 3.8	11.1 ± 2.1	0.751	9.2 ± 3.3	10.2 ± 2.5	0.002
Verbal fluency test	38.5 ± 7.9	40.3 ± 8.6	0.071	33.4 ± 9.6	35.2 ± 8.7	0.090

Abbreviations: VLT-R, verbal learning test-revised; VMT-R, visuospatial memory test-revised.

### Preoperative RDW and POCD risk

We firstly divided the RDW into two groups (≤14.7 vs. >14.7) according to the cut-off value of ROC. The univariate logistic regression indicated that preoperative RDW> 14.7 increased the risk of POCD (OR = 2.76, 95% CI: 1.32–5.16, *P*<0.001). Besides, the age > 65 years, the aortic plaque, carotid artery stenosis, cerebrovascular diseases, HDL-C, fasting plasma glucose, hs-CRP, TNF-α, white blood cell and surgery duration were also associated with the POCD risk. After adjustment of potential factors, the high preoperative RDW was still associated with an increased risk of POCD (OR = 2.52, 95% CI: 1.28–4.31, *P*<0.001, Table 3). Furthermore, the risk of POCD was also significantly raised by the aortic plaque (OR = 1.46, 95% CI: 1.06–2.74, *P*=0.037), cerebrovascular diseases (OR = 2.87, 95% CI: 1.43–4.22, *P*<0.001), high fasting plasma glucose level (OR = 1.119, 95% CI: 1.03–1.89, *P*=0.041), hs-CRP (OR = 1.26, 95% CI: 1.12–3.16, *P*=0.024) and long surgery duration (OR = 2.12, 95% CI: 1.13–2.89, *P*=0.001).

**Table 3:** The logistic regression of POCD-associated risk factors

Parameters	Univariate		Multivariate	
	OR (95%CI)	*P*	OR (95%CI)	*P*
Age > 65 years	1.23 (1.08–2.13)	0.035		
Aortic plaque	2.05 (1.28–3.28)	0.003	1.46 (1.06–2.74)	0.037
Carotid artery stenosis	1.77 (1.09–2.87)	0.020		
Cerebrovascular disease	3.26 (1.90–5.60)	<0.001	2.87 (1.43–4.22)	<0.001
HDL-C, mmol/dl	0.12 (0.06–0.76)	0.027		
Fasting plasma glucose, mmol/l	1.28 (1.12–2.64)	0.001	1.19 (1.03–1.89)	0.041
Hs-CRP, ng/l	2.01 (1.26–4.12)	0.008	1.26 (1.12–3.16)	0.024
TNF-α, ng/l	1.15 (1.07–2.74)	0.038		
White blood cell, ×10^9^/l	1.18 (1.06–2.36)	0.037		
Red cell distribution width (%) > 14.7	2.76 (1.32–5.16)	<0.001	2.52 (1.28–4.31)	<0.001
Duration of surgery	2.24 (1.38-3.93)	<0.001	2.12 (1.13-2.89)	0.001

## Discussion

The present study based on 21-day cognitive function assessment found that preoperative RDW was significantly higher in the POCD patients receiving CABG than in the non-POCD patients. The preoperative RDW had a moderate ability for diagnosis of 21-day cognitive dysfunction. The elevated preoperative RDW was associated with the risk of 21-day cognitive dysfunction, and this relationship was independent on other potential cofounding factors. As far as we know, this is the first report that assesses the relationship between RDW and POCD in patients receiving CABG. Our study provides a new biomarker for early diagnosis of POCD.

POCD is one of the most common complications after CABG and is mainly manifested as impairment of memory ability, abstraction ability, mental concentration, and knowledge analysis and applying ability, which seriously interferes with the rehabilitation and prognosis of coronary heart disease patients after CABG. As reported, POCD occurs in 65% of patients discharged after CABG surgery and in ∼40% in the months following surgery on average [22]. The 21-day POCD incidence of 27.1% in the present study is similar with previous reports [23–25]. RDW suggests the heterogeneity of red blood cell size, and reflects the relative number and function of red blood cells. The reference value of RDW ranges from 11.5to 14.5%. An increase in RDW indicates an increment in erythrocyte volume variability, which is common in patients with erythropoiesis disorders, severer erythrocyte destruction, or blood transfusion [26]. The inflammatory state and oxidative stress change the morphology, deforming force, and half-life of red blood cells, increase RDW *in vivo*, and cause changes in hemodynamic state [27]. RDW clinically influences the severity and prognosis of stroke, acute coronary syndrome, and other diseases [28,29]. Our study displays that preoperative RDW is associated with cognitive decline in CHD patients undergoing CABG. The reason can be partly attributed to inflammation response. The inflammation factors and inflammatory cells are usually at high levels in people who have ever received surgery. Neuroinflammation happens more easily in stress situations and leads to the impairment of cognitive function [30]. The neuroinflammatory mechanism of POCD includes several aspects. One aspect involves inflammatory cells: Astrocytes block nerve regeneration and release inflammatory factors. Activated microglia release neurotoxic substances, damage neurons, and exert a long-term effect with chronic continuous activation [31]. Inflammation in the central nervous system tends to infiltrate peripheral inflammatory cells, such as lymphocytes and monocytes, which affect cognitive function independently. Another aspect concerns inflammatory factors. Interleukin (IL)-1 can promote the release of neurotoxic substances and damage the formation of hippocampal long-term potentiation (LTP) through reactive oxygen species (ROS), mitogen activated protein kinase (MAPK), and other pathways. TNF-α can promote excitatory neurotoxic injury of glutamine, easily infiltrate peripheral inflammatory factors, and expand the cascade inflammatory reaction of the nervous system [32]. Il-6 can inhibit the occurrence of LTP and hippocampal meristem and cause its morphological changes [33]. The effects of inflammatory cytokines have been demonstrated in animal experiments *in vitro*. Besides, inflammatory cytokines work together to damage neurons. RDW is closely related to inflammatory responses. Lippi et al. first reported that RDW was independently associated with hs-CRP level [34]. Our study also indicates preoperative RDW is positively related to hs-CRP. It is well-known that hs-CRP is a typical downstream inflammatory marker and participates in immune cell chemotaxis, macrophage phagocytosis, platelet activation and complement activation [35]. Moreover, inflammatory factors inhibit the expression of erythropoietin genes and the proliferation of erythroid progenitor cells, and downregulate erythropoietin and its receptors, thereby shortening the lifespan of red blood cells [36]. High RDW reduces the production conditions of red blood cells, leading to an increased fragmentation of red blood cells, including an increased fragility. The increase in RDW may reflect an underlying inflammatory response that can shorten the survival of red blood cells and affect iron metabolism.

The other mechanism can be oxidative stress. Reportedly, RDW is associated with serum selenium, which together with inflammation predicts RDW in older women [37]. Under normal circumstances, the body maintains the balance between the oxidative system and the antioxidant system, but this balance will be quickly broken once the body is stimulated by trauma, surgery, or other external stimuli. Under excessive stress conditions, cells will produce abundant reactive oxygen radicals [38]. The excessive free radicals act on lipids *in vivo*, which leads to lipid peroxidation. The final product of oxidation is malondialdehyde with strong biological toxicity, which can destroy the structures and states of proteins and other macromolecules in cells [39]. While the body adapts to and avoids this kind of change caused by free radicals, it has evolved a balanced system to neutralize the excessive free radicals, including antioxidant enzymes of the antioxidant system, such as glutathione peroxidase (GPxs), superoxide dismutase (SOD) and the antioxidant enzymes, such as their common balance of oxidation reduction and the integrity of components [40]. Therefore, malondialdehyde and SOD can be used as markers of oxidative stress. Due to the high blood flow, high metabolism and high oxygen consumption, the brain is more prone to producing reactive oxygen radicals than other organs. At the same time, the antioxidant system in the brain is not developed. Once the oxidative stress reaction occurs, the excessive free radicals cannot be removed in time, thus reducing the resisting ability of the nervous system against external stimuli [41]. Furthermore, older people have a higher risk of developing neurological diseases because their nervous systems degrade during aging, making them more vulnerable to oxygen free radicals. When the brain experiences trauma, surgery, or other ischemic hypoxic stimulus, the microglia quickly causes excessive activation of the nervous system, releasing numerous inflammatory cytokines. At the same time, the free radicals produced by oxidative stress can accelerate inflammatory factors of cell damage and thereby cause neuronal injury and apoptosis, which influence cognitive function, leading to POCD [42]. Considering the relationship between RDW and oxidative stress, we think it reasonable that RDW is associated with POCD.

Our study has several limitations. Firstwe selected the 21-day occurrence rate of POCD, which was a short-term follow-up outcome, so longer follow-up is required to validate our findings. Secondldthe present study is targeted at CHD patients receiving CABG, so our results need to be confirmed in other population settings. Thirdly, we presents the association of preoperative RDW with POCD, but we have not explored the molecular mechanism. Finally, the sample size is relatively small, which calls for studies with larger sample size and more rigorous design to confirm our results. Besides, some variables including antifibrinolytics and bleeding volume are not included, which may affect the results. We have assumed antifibrinolytics may have little effect because all patients have received this treatment.

In conclusion, preoperative RDW is significantly elevated in POCD patients receiving CABG. The elevated preoperative RDW is associated with an increased risk of POCD, and preoperative RDW can be an independent predictor of POCD. To prevent POCD, early intervention in clinic is required by high-RDW patients. Nevertheless, future research should focus on the specific molecular mechanism.

## Abbreviations

BMI: body mass index
CABG: coronary artery bypass grafting
CI: confidence interval
HDL-C: high density lipoprotein-cholesterol
hs-CRP: hypersensitive C reactive protein
LTP: long-term potentiation
MMSE: mini-mental state examination
OR: odds ratio
POCD: postoperative cognitive dysfunction
RDW: red blood cell distribution width
ROC: receiver operating characteristic
SAS: self-rating anxiety scale
SDS: self-rating depression scale
SOD: superoxide dismutase
